# Supplementary material for: Dilation of fusion pores by crowding of SNARE proteins
Source: eLife. 2017 Mar 27;6:e22964. doi: 10.7554/eLife.22964 (PMC5404929; doi:10.7554/eLife.22964)
Supplement: Figure 5—source data 1. — Multiple pairwise comparisons of the group means were performed using 1-way analysis of variance (ANOVA) and a multiple comparison test using Matlab. The zipped file includes a matlab file (Figure 5a_FusionPoresPerMin_vs_vNLPcopies.mat) containing the fusion rate data and the results of the ANOVA and multiple comparison tests. Three figures summarize the test results (Figure 5a_ANOVAtable.fig, Figure 5a_ANOVAboxplot.fig, and Figure 5a_multcompare.fig). The analysis procedure and the results are explained in the pdf file Figure 5a_FusionRateAnalysis_summary.pdf. DOI: http://dx.doi.org/10.7554/eLife.22964.013 [file elife-22964-fig5-data1.zip › Fig5-SourceData1/Fig5a_FusionRateAnalysis_summary.pdf]

Dilation of fusion pores by crowding of SNARE proteins

Zhenyong Wu, Oscar D. Bello, Sathish Thiyagarajan, Sarah M. Auclair, Wensi Vennekate, Shyam S. Krishnakumar, Ben O'Shaughnessy, and Erdem Karatekin

Fig.5—source data 1

Summary of Matlab analysis of fusion rate statistics for different NLP v-SNARE copy numbers

Perform a 1-way ANOVA to test if the means of groups (different SNARE copy numbers) are different

Then use the ANOVA results to do pairwise comparisons using ‘multcompare’

```
% copy pores/min data (one value per cell) from excel to matlab. Name PperMinvNLP00, etc. These are vectors of different lengths (different numbers of cells), so fill with NaNs to 40 elements each (the longest vector, vNLP30 has 40 observations).
>> PperMinvNLP15(24:40)=NaN;
>> PperMinvNLP08(27:40)=NaN;
>> PperMinvNLP04(21:40)=NaN;
>> PperMinvNLP01(33:40)=NaN;
>> PperMinvNLP00(27:40)=NaN;

% combine into a matrix. Each column is a different group.
>> Frates = [PperMinvNLP00, PperMinvNLP01, PperMinvNLP04, PperMinvNLP08, PperMinvNLP15,
PperMinvNLP30];

% give meaningful names the groups (“levels”)
>> groups={'eNLP', 'vNLP1', 'vNLP4', 'vNLP8', 'vNLP15', 'vNLP30'};

% Perform 1-way ANOVA
>> [p,tbl,stats] = anova1(Frates, groups);
```

ANOVA Table

Source	SS	df	MS	F	Prob>F
Groups	1.10873	5	0.22175	5.38	0.0001
Error	6.63163	161	0.04119		
Total	7.74036	166			

rejects the null hypothesis that all group means are equal.

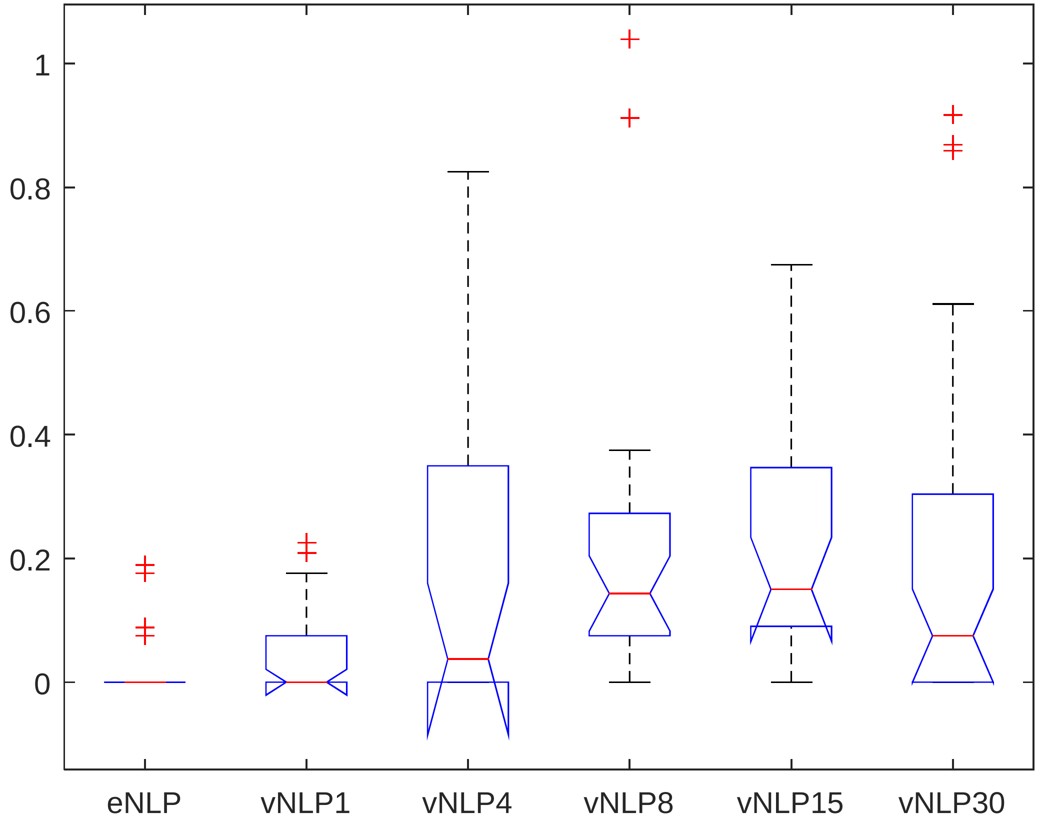

```
% we saved the statistics in the structure stats
>> stats
stats =
    gnames: {6x1 cell}
         n: [26 32 20 26 23 40]
    source: 'anova1'
     means: [0.0232 6.6936e-04 0.1700 0.2058 0.2324 0.1921]
        df: 161
         s: 0.2009
```

```
% Perform a multiple comparison test to determine which groups are different than the others in terms of mean fusion rate. Use the function multcompare, which accepts stats as an input argument.

>> multcompare(stats)
ans =

    1.0000    2.0000   -0.1696   -0.0169    0.1358    0.9996
    1.0000    3.0000   -0.3188   -0.1468    0.0252    0.1451
    1.0000    4.0000   -0.3430   -0.1826   -0.0222    0.0149
    1.0000    5.0000   -0.3747   -0.2092   -0.0436    0.0043
    1.0000    6.0000   -0.3146   -0.1689   -0.0232    0.0123
    2.0000    3.0000   -0.2947   -0.1299    0.0350    0.2171
    2.0000    4.0000   -0.3184   -0.1657   -0.0130    0.0244
    2.0000    5.0000   -0.3503   -0.1922   -0.0341    0.0070
    2.0000    6.0000   -0.2891   -0.1519   -0.0148    0.0199
    3.0000    4.0000   -0.2078   -0.0358    0.1362    0.9915
    3.0000    5.0000   -0.2392   -0.0624    0.1145    0.9165
    3.0000    6.0000   -0.1804   -0.0220    0.1363    0.9987
    4.0000    5.0000   -0.1921   -0.0266    0.1390    0.9975
    4.0000    6.0000   -0.1320    0.0137    0.1594    0.9998
    5.0000    6.0000   -0.1110    0.0403    0.1917    0.9742
```

Each row of the matrix contains the result of one paired comparison test. Columns 1 and 2 contain the indices of the two samples being compared. Column 3 contains the lower confidence interval, column 4 contains the estimate, and column 5 contains the upper confidence interval. Column 6 contains the p-value for the hypothesis test that the corresponding mean difference is not equal to 0.

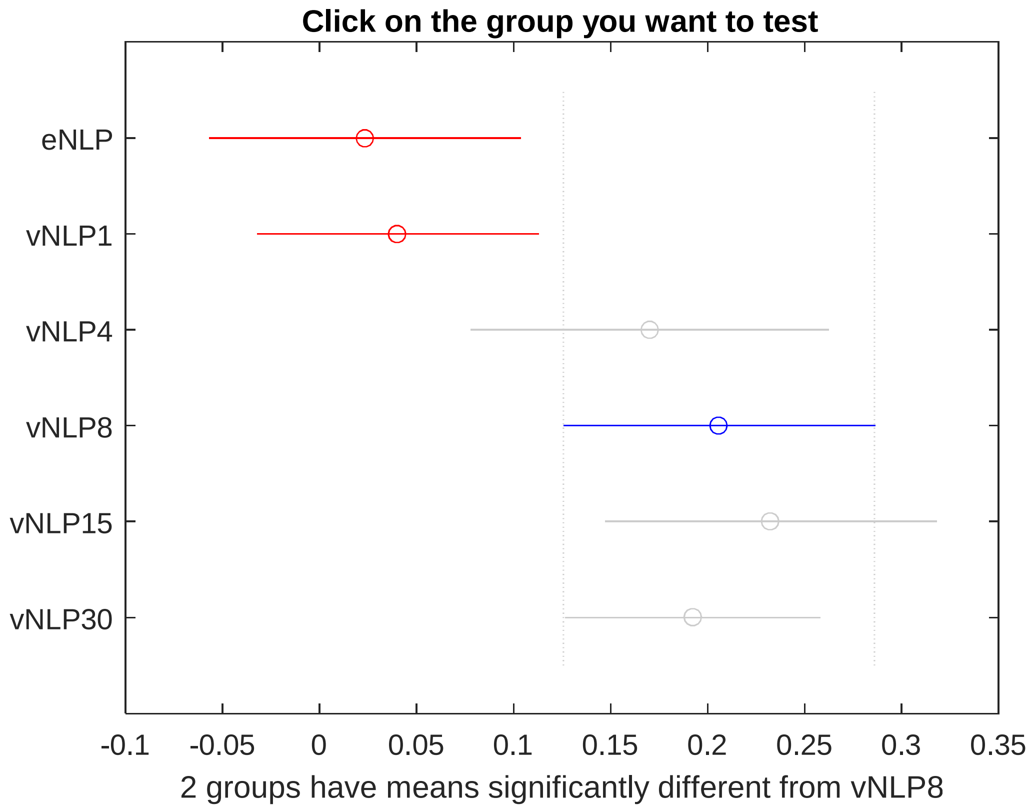

We also get an interactive plot.
There are no significant differences in the means among the groups vNLP4, vNLP8, vNLP15, and vNLP30.

In this example, clicking on vNLP8 (turns blue), we see pairwise comparisons with the other groups. Only eNLP and vNLP1 have different means.(red).

eNLP is not different than vNLP1 and vNLP4.
vNLP1 is not different than eNLP and vNLP4
vNLP4 is not different from any other.
vNLP8, 15, 30 are not different form one another, but different than eNLP and vNLP1
